# Textual analysis of sugar industry influence on the World Health Organization’s 2015 sugars intake guideline

**DOI:** 10.2471/BLT.15.165852

**Published:** 2016-05-12

**Authors:** David Stuckler, Aaron Reeves, Rachel Loopstra, Martin McKee

**Affiliations:** a Department of Sociology, University of Oxford, Manor Road Building, Oxford, OX1 3UQ, England.; b International Inequalities Institute, London School of Economics and Political Science, London, England.; c Department of Public Health & Policy, London School of Hygiene & Tropical Medicine, London, England.

## Abstract

**Objective:**

To determine whether sugar industry-related organizations influenced textual changes between the draft and final versions of the World Health Organization’s (WHO’s) 2015 guideline *Sugars intake for adults and children*.

**Methods:**

Stakeholder consultation submissions on the draft guideline from seven sugar industry-related and 10 public health organizations were assessed using the Wordscores program. Document scores were rescaled using the Martin–Vanberg transformation to improve comparability. Draft and final guidelines were compared to identify changes influenced by the sugar industry and public health organizations.

**Findings:**

There was a small shift in transformed Wordscores score between the draft and final guidelines, from 0.25 to 0.24, towards the industry position. The change was linked to increased use of the word “low” to describe the quality of the evidence, consistent with industry arguments. There was also a shift from use of the word “consumption” to “intake”, irrespective of policy position. Scores for World Sugar Research Organisation and Sugar Nutrition UK submissions ( 0.11 and 0.18, respectively) represented strong pro-industry positions and scores for European Public Health Alliance and Wemos submissions (1.00 and 0.88, respectively) represented the strongest public health positions. Industry tactics included challenging the quality of the evidence, distinguishing between different types of sugar and advocating harm reduction.

**Conclusion:**

There was little change between draft and final versions of the WHO sugars intake guideline 2015, following industry consultation. The main change was linked to emphasizing the low quality of the evidence on sugar’s adverse effects. Guideline development appeared relatively resistant to industry influence at the stakeholder consultation stage.

## Résumé

**Objectif:**

Déterminer si les organisations liées au secteur sucrier ont influencé les modifications textuelles entre la version provisoire et la version finale de la directive de 2015 de l'Organisation mondiale de la Santé (OMS) *Apport en sucres chez l'adulte et l'enfant*.

**Méthodes:**

Les propositions issues de consultations avec les parties prenantes (7 organisations liées au secteur sucrier et 10 organismes de santé publique) au sujet de la version provisoire de la directive ont été évaluées à l'aide du programme Wordscores. Les scores des documents ont été réajustés suivant la procédure de transformation de Martin–Vanberg afin d'en améliorer la comparabilité. La version provisoire et la version finale de la directive ont été comparées afin de repérer les modifications influencées par l'industrie sucrière et les organismes de santé publique.

**Résultats:**

On a observé un léger changement du score Wordscores transformé (de 0,25 à 0,24) entre la version provisoire et la version finale, en faveur de la position de l'industrie. Ce changement était lié à l'emploi plus fréquent du mot « mauvaise » pour décrire la qualité des éléments de preuve, ce qui concordait avec les arguments de l'industrie. Un autre changement concernait l'utilisation du mot « apport » au lieu de « consommation », indépendamment de la position politique. Les scores des propositions des organisations *World Sugar Research Organisation* et *Sugar Nutrition UK* (respectivement 0,11 et 0,18) représentaient de fortes positions pro-industrie et les scores des propositions de la *European Public Health Alliance* et de la fondation Wemos (respectivement 1,00 et 0,88) représentaient les plus fortes positions du secteur de la santé publique. L'industrie sucrière a usé de tactiques telles que la remise en question de la qualité des éléments de preuve, la distinction entre les différents types de sucres et la mise en avant de la réduction des méfaits.

**Conclusion:**

Peu de changements ont été constatés entre la version provisoire et la version finale de la directive de 2015 de l'OMS sur l'apport en sucres suite à la consultation des organisations du secteur. Le principal changement concernait la mise en avant de la mauvaise qualité des preuves sur les effets néfastes du sucre. L'élaboration de la directive a relativement bien résisté à l'influence de l'industrie sucrière lors de la phase de consultation des parties prenantes.

## Resumen

**Objetivo:**

Determinar si las organizaciones relacionadas con la industria azucarera han influenciado en las modificaciones de texto entre el borrador y las versiones definitivas de la directriz *Ingesta de azúcares para adultos y niños* de 2015 de la Organización Mundial de la Salud (OMS).

**Métodos:**

Se evaluaron las consultas de las partes interesadas sobre el borrador de la directriz enviadas por 7 organizaciones relacionadas con la industria azucarera y 10 organizaciones de salud pública utilizando el programa Wordscores. Los resultados del documento se clasificaron utilizando la transformación de Martin y Vanberg para mejorar la comparación. Se compararon el borrador y las directrices definitivas para identificar los cambios influenciados por la industria azucarera y las organizaciones de salud pública.

**Resultados:**

Se detectó un ligero cambio en la puntuación de Wordscores transformada entre el borrador y las directrices definitivas, de 0,25 a 0,24 a favor de la industria. El cambio se relacionaba con el aumento del uso de la palabra “baja” para describir la calidad de la prueba, de forma coherente con los argumentos de la industria. También se encontró un cambio del uso de la palabra “consumo” a “ingesta”, independientemente de la postura política. Los resultados de la Organización Mundial de Investigación del Azúcar y Sugar Nutrition UK (0,11 y 0,18 respectivamente) representaron posturas muy favorables a la industria y los resultados de Alianza Europea de Salud Pública y Wemos (1,00 y 0,88 respectivamente) representaron las posturas más favorables a la salud pública. Entre las tácticas industriales se encontraban la obstaculización de la calidad de las pruebas, la distinción entre distintos tipos de azúcares y la defensa de la reducción de daños.

**Conclusión:**

Apenas se detectaron cambios entre el borrador y las versiones definitivas de la directriz sobre la ingesta de azúcares de 2015 de la OMS, tras la consulta a la industria. El principal cambio estaba relacionado con la enfatización de la escasa calidad de las pruebas sobre los efectos perjudiciales del azúcar. El desarrollo de la directriz se mostró relativamente resistente a la influencia de la industria en la etapa de consulta a las partes interesadas.

## ملخص

**الغرض:**

تحديد ما إذا كانت المنظمات المرتبطة بصناعة السكر أثرت على التغييرات النصية بين المسودة والإصدارات النهائية للمبدأ التوجيهي الخاص بمنظمة الصحة العالمية لعام 2015 بشأن تناول السكريات للبالغين والأطفال.

**الطريقة:**

تم تقييم الطلبات المقدمة نتيجة التشاور مع الجهات المعنية عن مسودة المبدأ التوجيهي من 7 منظمات مرتبطة بصناعة السكر و10 منظمات صحية عامة باستخدام برنامج Wordscores. تمت إعادة صياغة نتائج المستندات باستخدام طريقة تحويل النصوص Martin–Vanberg لتحسين إمكانية المقارنة. وتمت مقارنة المسودة والإصدار النهائي من المبادئ التوجيهية للتعرّف على التغييرات المتأثرة من جانب صناعة السكر ومنظمات الصحة العامة.

**النتائج:**

كان هناك تغيير طفيف في نتائج Wordscores **التي تم **تحويلها ما بين المسودة والإصدار النهائي من المبادئ التوجيهية، من 0.25 إلى 0.24، في اتجاه موقف الصناعة. كان التغيير مرتبطًا بالاستخدام الزائد لكلمة "منخفض" لوصف جودة الدليل، بالتوافق مع النقاشات السائدة في قطاع الصناعة. كان هناك أيضًا تغيير في استخدام كلمة "استهلاك" واستبدالها بكلمة "تناول" بصرف النظر عن موقف السياسات. كانت نتائج الطلبات المقدمة من منظمة بحوث السكر العالمية ومنظمة التغذية بالسكر في المملكة المتحدة (0.11 و0.18 على التوالي) تمثل المواقف القوية المؤيدة للصناعة، وكانت نتائج الطلبات المقدمة من التحالف الأوروبي للصحة العامة وWemos (بواقع 1.00 و0.88 على التوالي) تمثل أقوى مواقف الصحة العامة. تضمنت أساليب الصناعة الاعتراض على جودة الأدلة، والتمييز بين أنواع مختلفة من السكر، والسعي إلى التقليل من الأضرار.

**الاستنتاج:**

كان هناك تغيير طفيف بين المسودة والإصدارات النهائية للمبدأ التوجيهي الصادر عن منظمة الصحة العالمية عام 2015 بشأن تناول السكريات، وذلك في أعقاب المشاورات مع القائمين على الصناعة. كان التغيير الأساسي مرتبطًا بالتأكيد على تدني الجودة النوعية للأدلة المتعلقة بالآثار السلبية للسكر. وبدا أن عملية إعداد المبادئ التوجيهية مقاومة نسبيًا لتأثير القائمين على الصناعة في مرحلة التشاور مع الجهات المعنية.

## 摘要

**目的:**

旨在明确制糖业相关组织是否在世界卫生组织 (WHO) 2015 年度《成人和儿童糖摄入量指南》的草案到定稿期间影响文本更改。

**方法:**

我们使用 Wordscores 程序评估了 7 家制糖业相关组织和 10 家公共卫生组织对指南草案的利益相关方协商意见书，并且采用 Martin–Vanberg 转换法重新调整了文档匹配度，以期提高可比性。对指南草案和定稿进行了比较，进而确定受制糖业和公共卫生组织影响的变更。

**结果:**

转换后的指南草案和定稿的 Wordscores 匹配度有些变化，其行业地位从 0.25 变为 0.24。 该变化与为了描述证据质量而增加使用“低”字有关，这与行业论点一致。另一个转变为从“消耗”一词转为“摄入”，不论在何种政策主张下。世界糖业研究组织 (World Sugar Research Organisation) 和英国糖营养协会 (Sugar Nutrition UK) 意见书的匹配度（分别为 0.11 和 0.18）代表较为靠前的行业地位，欧洲公共卫生联盟 (European Public Health Alliance) 和 Wemos 意见书的匹配度（分别为 1.00 和 0.88）代表最高的公共卫生行业地位。行业策略包括验证证据质量、区分不同糖种类和倡导降低危害。

**结论:**

经行业协商后， 2015 年世界卫生组织糖摄入量指南的草案和定稿之间基本没有变更。主要变化是为了强调关于糖的不良影响的证据质量很差。在利益相关方协商阶段，指南的编制基本表现出不受行业影响。

## Резюме

**Цель:**

Определить, оказали ли влияние организации, связанные с сахарной промышленностью, на текстуальные различия между черновым и окончательным вариантом руководящих принципов Всемирной организации здравоохранения Sugars intake for adults and children (Использование сахаров взрослыми и детьми) 2015 года.

**Методы:**

С помощью программы Wordscores была осуществлена оценка представлений, направленных после консультации с заинтересованными лицами по черновому варианту руководящих принципов. Представления были направлены от 7 организаций, связанных с сахарной промышленностью, и 10 организаций общественного здравоохранения. Показатели анализа документов были нормализованы с помощью преобразования Мартина-Ванберга для улучшения сопоставимости. Черновой и окончательный варианты руководства сравнили для того, чтобы определить, какие изменения были вызваны организациями сахарной промышленности и общественного здравоохранения.

**Результаты:**

Черновой и окончательный варианты руководства незначительно изменились в плане результатов анализа Wordscores, подвергнутых преобразованию, — с 0,25 до 0,24 в сторону сближения с позицией представителей сахарной промышленности. Это изменение было связано с увеличением употребления слова «low» (низкий) для описания качества фактических данных, приводимых против доводов представителей промышленности. Также слово «consumption» (потребление) было заменено словом «intake» (использование) сторонниками обеих позиций по политике. Судя по показателям оценки представлений, Всемирная организация исследования сахара и Sugar Nutrition UK были наиболее убежденными сторонниками позиции сахарной промышленности (0,11 и 0,18 соответственно), а позиции общественного здравоохранения сильнее других поддерживали Европейский альянс общественного здравоохранения и Wemos (1,00 и 0,88 соответственно). Тактика представителей промышленности включала оспаривание качества фактических данных, проведение различения между видами сахара и пропагандирование мер по снижению вреда.

**Вывод:**

Различия, возникшие в результате консультации представителей промышленности, между черновым и оригинальным вариантами руководства Всемирной организации здравоохранения 2015 года, касающимися потребления сахаров, были незначительными. Основное изменение было связано с усилением акцента на низком качестве фактических данных, подтверждающих неблагоприятное действие сахара. Процесс разработки руководства оказался относительно устойчивым к влиянию промышленности на этапе консультации с заинтересованными лицами.

## Introduction

In 2013, the World Health Organization (WHO) began the process of updating its guideline on sugars intake for adults and children. Sugar consumption has been a contested area of global public health for at least four decades, initially stimulated by Yudkin’s 1972 classic, *Pure, white and deadly*,^1^ and Cleaves’ 1974 book, *The saccharine disease*,^2^ which linked dietary sugar intake to obesity, diabetes and other noncommunicable diseases. At the time, the arguments in these books were met with great scepticism but, since then, supporting evidence has grown and now includes the findings of randomized controlled trials and meta-analyses. Early studies linked sugar intake to dental caries and obesity,^3^^,^^4^ particularly in children,^5^ and recent evidence supports an association with conditions such as diabetes and nonalcoholic fatty liver disease.^6^^,^^7^ Nonetheless, discussions continue about the appropriate measure (i.e. grams or percentage of daily total energy intake), type (i.e. free versus added sugars) and saccharide structure (i.e. monosaccharide and disaccharide) of dietary sugars and whether these factors influence the risk to health. These debates complicate the process of developing guidelines.

Quite possibly, WHO is the most important public health agency responsible for developing global guidelines. One of its core functions is to ensure that evidence is used appropriately. In the past, WHO has produced guidelines on blood pressure, patient safety, mental health and substance abuse, among other topics. Several are contested because their recommendations conflict with the goals of multinational pharmaceutical companies or tobacco, alcohol, food or beverage companies.^8^^,^^9^ Historically, organizations linked to the sugar industry have attacked WHO’s recommendations on limiting sugar consumption. In 2003, WHO released a joint report with the Food and Agriculture Organization entitled *Diet, nutrition and the prevention of chronic diseases*^10^ which, for the first time, called for a reduction in sugar intake to under 10% of total dietary energy consumption. The Sugar Association wrote that it would “exercise every avenue available to expose the dubious nature” of WHO's report on diet and nutrition and would challenge WHO's funding from the United States of America, which was 406 million United States dollars in 2003.^11^ The association erroneously claimed that the report was written by selected experts and was not peer-reviewed and that industry did not have an opportunity to comment.^12^

Although previous attempts to influence WHO did not lead to a change in sugar recommendations, there have been concerns that vested interests would seek to influence the process of updating dietary guidelines.^13^^,^^14^ One recent analysis of documents from the sugar industry revealed that some companies had worked with government researchers to soften government recommendations in the United States on reducing sugar intake to help prevent cavities and had influenced dental research – one report stated that 80% of the industry’s recommendations had been adopted.^13^ Moreover, an investigation of the sugar industry in the United Kingdom of Great Britain and Northern Ireland found a tangled web of “connections between the sugar industry and nutrition experts”, which led to an explicit call for careful scrutiny of WHO’s new sugars intake guideline.^14^ Concerns about the participation of the sugar industry in WHO’s stakeholder consultations on the evidence underlying this guideline arose because, in the past, the tobacco industry has exploited such consultations to exert influence.^15^^,^^16^ Nevertheless, WHO’s process for developing guidelines, which is based on the second edition of the *WHO handbook for guideline development* (Box 1),^17^ is quite extensive and is regarded as being resistant to industry influence.

Box 1 **Development process for WHO’s sugars intake guideline, 2015**

Development of the 2015 guideline on sugars intake for adults and children by the World Health Organization (WHO) followed procedures outlined in the *WHO handbook for guideline development*, second edition.^17^^,^^18^ The guideline was developed by WHO’s Department of Nutrition for Health and Development in partnership with members of the WHO Secretariat. A strict process was followed to manage potential conflicts of interest. All experts participating in meetings had to declare their relevant interests in advance. These interests were then reviewed by the WHO Secretariat in consultation with the WHO Office of the Legal Counsel. A guideline development group and an external peer-review group were formed and public consultations were carried out.

**Guideline development group**

The guideline was developed by WHO’s Nutrition Guidance Expert Advisory Group’s Subgroup on Diet and Health. Members of the subgroup included experts who had previously participated in expert consultations for WHO, who were members of WHO’s expert advisory panels or who had been identified through an open call for experts. Representatives of commercial organizations were not invited to participate because their membership of a WHO guideline group was considered inappropriate due to actual, potential or perceived conflicts of interest. The subgroup reviewed and assessed the quality of the evidence available on the existing and new sugars guidelines and advised WHO on selecting outcomes important for decision-making and on interpreting the evidence used to produce recommendations. The overall quality of the evidence was assessed by systematic review and using the GRADE (grading of recommendations assessment, development and evaluation) approach.

**External peer-review group**

The WHO Secretariat selected external peer-reviewers from among representatives of public institutions that are members of WHO’s Global Network of Institutions for Scientific Advice on Nutrition, experts in the subject matter and other stakeholders, such as medical practitioners and scientific journal editors. The resulting peer-review group was asked to examine the draft guideline and identify any errors or missing information.

**Public consultations**

The initial public consultation asked for comments on the scope of the guideline and on specific research questions. The request was posted on the website of WHO’s Department of Nutrition for Health and Development and disseminated through the department’s mailing list and the mailing list of the United Nations Standing Committee on Nutrition. A second public consultation was carried out to obtain comments on the draft guideline before it was finalized. Contributors were contacted through the same two mailing lists.

To determine whether the sugar industry was able to influence the recent updating of WHO’s sugars intake guideline, we evaluated the textual content of the updated guideline using automated content analysis, a tool that has recently been used to quantify the influence of the tobacco lobby on policy.^15^ We determined whether WHO’s draft guideline had shifted towards a position that favoured the sugar industry by analysing the change in content of the guideline with reference to documents submitted during stakeholder consultations.

## Methods

Between 5 March and 31 March 2014, WHO consulted stakeholders on the new sugars intake guideline. For this analysis, we obtained submissions from organizations linked to the sugar industry and from leading public health organizations that were made publicly available by the organizations themselves. The ultimate selection of sources was the sole responsibility of the authors. Organizations identified as having potential links to the sugar industry were: the World Sugar Research Organisation; Food Industry Asia; the International Food & Beverage Alliance; Nestlé; Sugar Nutrition UK”; Kenniscentrum suiker & voeding; and Wirtschaftliche Vereinigung Zucker. Those identified as having public health interests were: the Centre for Science in the Public Interest; the United Kingdom Health Forum; the European consumers organization BEUC; the International Baby Food Action Network; the Mexican nongovernmental organization El Poder del Consumidor; the Dutch nongovernmental organization Wemos; the World Dental Federation; the World Obesity Federation; and the European Public Health Alliance.

We compared the policy positions taken by stakeholders with the content of WHO’s evolving guideline using the scaling algorithm Wordscores (StataCorp. LP, College Station, United States).^19^^,^^20^ Wordscores infers policy positions in new documents – so-called virgin texts – by calculating scores for these documents based on the scores of reference texts. Scores are derived from the frequency of words in a document (relative to the total number of words) on the assumption that stakeholders with different policy positions will use wording that reflects their ideology or stance (Box 2). For example, compared with public health bodies, the tobacco industry more frequently invokes arguments about the economy and business.^26^

Box 2 **Wordscores textual analysis**

With the textual analysis program Wordscores, the relative frequency of a word *w* in a reference text *r*, denoted *F_wr_*, is used to compute the conditional probability that we are reading text *r* if we are reading the word *w*. This probability is then used to derive a score, *S_w_*, for each word *w* that is the weighted average of the scores for all the reference texts in which the word *w* appears – the scores are weighted by the calculated conditional probability for each text. Then, the word scores are used to compute an overall document score for each virgin text *v*, denoted *ω_v_*, which is the sum of the scores for all the words contained in it weighted by their relative frequency *F_wv_*:

ω*_v_* = ∑*_w_ (F_wv_ . S_w_).*

This approach has previously been validated for political texts and speeches on economic policy.^21^^,^^22^ In the health field, it has been applied to European Commission documents.^23^ One limitation of Wordscores is that the estimated word scores of virgin texts are not directly comparable to the word scores of reference texts. Since reference texts tend to have many nondiscriminating words in common, their word scores tend to be pulled towards the middle of the scale.^24^ To compensate, word scores can be rescaled using the Martin–Vanberg transformation.^25^ Positions for both the reference and virgin texts are estimated and the most extreme positions observed among the calculated word scores are used to rescale the document scores for each virgin text, such that the document score becomes:

(ω*_v_* - ω*_1_*)/(ω*_1_* - ω*_2_*),

where ω_v_ again represents the raw document score for each virgin text and ω_1_ and ω_2_ represent the document scores for the virgin texts with the most extreme values.

First, we converted documents from portable document format into more manageable text files and manually removed superfluous information, including the names of interest groups, headers and footers, contact details and citations. Then we created a frequency matrix using the program JFreq in the programming language R,^27^ which replaced words by their roots and removed stop words, numbers and symbols. Second, we assigned scores to the reference texts – these served as the basis for classifying virgin texts. In view of the known polarization of opinion between public health and sugar industry-related organizations, we devised a scale using texts from the European Public Health Alliance and Wemos as reference texts representing the public health position and texts from the World Sugar Research Organisation and Nestlé as reference texts representing the industry position. In effect, the content of these four texts represented the two ends of a scale on which all other documents could be positioned, with each document’s position reflecting the opinion being expressed. In addition, we rescaled the scores using the Martin–Vanberg transformation, which compensates for the tendency for nondiscriminating words to pull scores towards the middle of the scale (Box 2).^25^ Transformed scores were calculated with 95% confidence intervals based on the standard deviations in score frequencies across documents.^19^ All scores were calculated using Stata version 13.0 (StataCorp. LP, College Station, United States). In the final step, we assessed changes in WHO’s new sugars intake guideline by comparing the scores of the draft guideline published in March 2014 and of the final version released in March 2015. As the choice of reference texts can potentially influence results, we checked the robustness of our selection by using all 10 texts from public health organizations (including WHO) to represent the public health position and all seven texts from sugar industry-related organizations to represent the industry position.

## Results

Overall, the most frequently used words relevant to sugar intake in submissions from the 17 sugar industry-related and public health organizations were “intake”, “sugars”, “dental” and “caries”. There was little mention of economics or business in either WHO’s final guideline or stakeholders’ submissions, apart from language about consumers, which comprised less than 0.2% (8/5239) of all words in WHO’s final guideline. The word “dental” (frequency: 1.3%; 67/5239) featured more prominently in WHO’s guideline than did the acronym “NCDs” for noncommunicable diseases (frequency: 0.4%; 21/5239), the word “obesity” (frequency: 0.3%; 14/5239), the word “diabetes” (frequency: 0.1%; 3/5239) or the acronym “CVD” for cardiovascular disease (frequency: 0.1%; 3/5239). Children were discussed more often than adults in WHO’s documentation: the frequency of words associated with children was 0.7% (39/5239), whereas the frequency of words associated with adults was 0.5% (25/5239). Children were referred to more frequently in stakeholders’ submissions. The word “physical” (with reference to physical activity) was mentioned in four of the seven submissions from sugar industry-related organizations but in only one submission from a public health organization (the Centre for Science in the Public Interest). Sugar industry-related organizations appeared to be emphasizing energy expenditure through physical activity in preference to energy intake from food.^28^

### Draft versus final guideline

The final column in Table 1 shows the rescaled, transformed, Wordscores scores for submissions from sugar industry-related and public health organizations and for the draft and final versions of WHO’s new sugars intake guideline. The score changed little between the draft and the final guideline: from 0.25 to 0.24. In addition, we disaggregated the textual changes between draft and final guidelines. As shown in Table 2, there was a marked shift from usage of the word “consumption” to that of the word “intake”, which occurred irrespective of the text’s source. Furthermore, usage of the words “low” and “moderate” increased substantially, which reflected the addition of descriptors about the weak quality of the evidence. The summary clauses in Box 3 illustrate this change.

**Table 1:** Textual analysis of consultation submissions and WHO’s sugars intake guideline, 2015

Document	Total no. of words	No. of unique words	Raw Wordscores^a^ score (SE)	Martin–Vanberg transformed^b^ Wordscores score		Rescaled transformed Wordscores score^c^
				Value (SE)	95% CI	
**Consultation submissions from sugar industry-related organizations**						
World Sugar Research Organisation	2703	803	0.32 (0.01)	0.03 (0.02)	−0.02 to 0.08	0.11
Food Industry Asia	1146	305	0.39 (0.01)	0.35 (0.04)	0.27 to 0.43	0.25
International Food & Beverage Alliance	709	241	0.37 (0.01)	0.29 (0.05)	0.19 to 0.39	0.23
Nestlé	524	222	0.27 (0.01)	−0.23 (0.05)	−0.32 to −0.13	0.00
Sugar Nutrition UK	2346	442	0.35 (0.01)	0.18 (0.03)	0.13 to 0.24	0.18
Kenniscentrum suiker & voeding	1958	331	0.36 (0.01)	0.22 (0.03)	0.16 to 0.27	0.19
Wirtschaftliche Vereinigung Zucker	2795	453	0.35 (0.00)	0.17 (0.02)	0.12 to 0.21	0.17
**Consultation submissions from public health organizations**						
Centre for Science in the Public Interest	1400	331	0.32 (0.01)	−0.01 (0.03)	−0.07 to 0.06	0.10
United Kingdom Health Forum	2539	445	0.43 (0.01)	0.55 (0.03)	0.50 to 0.60	0.34
BEUC^d^	1468	337	0.37 (0.01)	0.24 (0.03)	0.17 to 0.31	0.21
International Baby Food Action Network	2430	456	0.34 (0.01)	0.12 (0.03)	0.07 to 0.18	0.15
El Poder del Consumidor^e^	849	235	0.38 (0.01)	0.30 (0.04)	0.21 to 0.39	0.23
Wemos^f^	820	335	0.68 (0.01)	1.78 (0.04)	1.69 to 1.86	0.88
World Dental Federation	1969	391	0.35 (0.01)	0.14 (0.03)	0.08 to 0.20	0.16
World Obesity Federation	173	88	0.38 (0.02)	0.30 (0.10)	0.10 to 0.49	0.23
European Public Health Alliance	1088	515	0.73 (0.01)	2.04 (0.04)	1.97 to 2.12	1.00
**WHO sugars intake guideline, 2015**						
Draft version	5643	537	0.38 (0.00)	0.34 (0.02)	0.30 to 0.37	0.25
Final version	6447	550	0.38 (0.00)	0.32 (0.02)	0.29 to 0.35	0.24

CI: confidence interval; SE: standard error; WHO: World Health Organization.

^a^ The Wordscores program calculates scores for documents based on the scores of reference documents by assessing the frequency of words in the documents after words were replaced by their roots and stop words, numbers and symbols were removed.

^b^ The Martin–Vanberg transformation compensates for the tendency for nondiscriminating words to pull scores towards the middle of the scale.

^c^ The Wordscores scores obtained after the Martin–Vanberg transformation were rescaled such that the text from Nestlé had a score of 0 and that from the European Public Health Alliance had a score of 1.

^d^ BEUC is an international European consumers organization.

^e^ El Poder del Consumidor is a Mexican nongovernmental organization.

^f^ Wemos is a Dutch nongovernmental organization.

**Table 2:** Change in word usage between draft and final versions of the WHO’s sugars intake guideline, 2015

Word	Word count, no.	
	Draft guideline	Final guideline
**Words with a large increase in usage**		
Intake	104	148
Low	9	22
Quality	16	30
Effect	5	17
Moderate	4	15
**Words with a large decrease in usage**		
Consumption	19	2
Obesity	17	14
Caries	65	62
Policy	9	5

WHO: World Health Organization.

Box 3 **Textual changes between draft and final versions of WHO’s sugars intake guideline, 2015**

The following textual changes between the draft and final versions of the World Health Organization’s 2015 sugars intake guideline illustrate: (i) increased usage of the word “low”, linked to the addition of descriptors about the weak quality of the evidence; (ii) increased usage of the word “intake”; and (iii) the use of more specific descriptions of the types of sugars.

The draft version of the guideline included the recommendations:

(1a) The recommendation to limit sugars intake to less than 10% of total energy is based on observational studies with dental caries as an outcome.

(2a) The recommendation to further limit sugars intake to less than 5% of total energy is based on ecological studies in which a linear relationship between sugars intake and dental caries was observed.

In the final version of the guideline, these were amended to (changes in italics):

(1b) The recommendation to limit *free* sugars intake to less than 10% of total energy *intake* is based on *moderate quality evidence from* observational studies *of* dental caries.

(2b) The recommendation to further limit *free* sugars intake to less than 5% of total energy *intake* is based on *very low quality evidence from* ecological studies in which a *positive dose–response* relationship between *free* sugars intake and dental caries was observed *at free* sugars intake *of* less than 5% of total energy *intake*.

To assess the robustness of our findings, we included all documents from sugar-industry related and public health organizations as reference documents, as described earlier. Although none of the main results were changed, the submission by the Centre for Science in the Public Interest shifted to being closer to the public health position when it was included as a reference text. In addition, we removed any words containing the word “sugar”, as they were relatively common in all documents. This did not alter the direction of the findings because use of these words did not discriminate between policy positions.

### Textual analysis

To identify the tactics used by the sugar industry, we carried out a qualitative analysis of its submissions. One tactic involved direct attacks on the implications of the evidence. The World Sugar Research Organisation’s submission claimed that there was no evidence to support WHO’s statement in the draft guideline that: “There is no harm associated with reducing the intake of free sugars to less than 5% of total energy.” Nevertheless, this statement was retained in the final document. Other industry submissions went further and argued that reducing sugar consumption could lead to higher fat intake.

A second tactic was to challenge the validity of the evidence. Several submissions from sugar industry-related organizations argued that the distinction between different types of sugar made by WHO was not scientific. For example, Sugar Nutrition UK stated that the “distinction between ‘free’, ‘added’ and ‘other’ sugars is not based on any sound scientific principles, given that it is impossible to analytically distinguish between sugars present naturally in a food and those which have been added during cooking or manufacturing”. Industry submissions also questioned the validity of the systematic reviews commissioned by WHO because they made recommendations about “free” sugars, whereas they had evaluated “total” sugars.

A third tactic was to undermine the strength of the evidence. For example, the World Sugar Research Organisation argued that: “The quantitative target of 10% cited in the Draft Guideline is based solely on observational cohort studies of dental caries in children. This is an inadequate basis for public health guidelines because of the certainty of confounding.” The organisation then argued that: “No recommendation can be made because of insufficient evidence” and cited the *WHO handbook for guideline development*. The International Food & Beverage Alliance took a similar approach, arguing that “there is insufficient scientific support that would justify the lowering of the current WHO guideline on consumption of free sugars to 5%”. Sugar Nutrition UK took a similar stance, stating that: “To use… ‘very low quality’ data as evidence to underpin a conditional recommendation of a ‘further reduction to below 5% of total energy’ contradicts WHO’s own guidelines.”

Finally, several industry submissions sought to place the emphasis on harm reduction. For example, the World Sugar Research Organisation argued that the risk of dental caries from sugar consumption can be mitigated; it stated: “Any effects of consumption of sugars will be attenuated by fluoride protection.” They further argued that

“available data do not allow the setting of an upper limit for intake of (added) sugars on the basis of a risk reduction for dental caries, as caries development related to consumption of sucrose and other cariogenic carbohydrates does not depend only on the amount of sugar consumed, but it is also influenced by frequency of consumption, oral hygiene, exposure to fluoride, and various other factors.”

Although the majority of sugar industry-related organizations opposed the guideline, Nestlé appeared more supportive, stating that: “We support [WHO’s] recommendation that you reduce the consumption of free sugars throughout the course of your life… [and] should not exceed 10% of your total energy intake, whether you are an adult or a child.” The company also argued for voluntary reductions in the sugar content of food and highlighted its commitment to reducing the sugar content of children’s cereals to 9 g or less per serving by 2015.

## Discussion

We found relatively little change overall in WHO’s 2015 guideline on sugars intake for adults and children from before to after the stakeholder consultation in 2014 despite strenuous efforts by industry to modify it. The changes observed were linked to increased use of the word “low” to describe the quality of the evidence and of the word “moderate” to describe the quality of observational cohort studies. Although the sugar industry used several tactics to undermine the guideline’s recommendations, their development was not substantially affected.

As with any statistical analysis, this study has several important limitations. First, because we examined differences between the draft and final versions of the guideline rather than the details of the texts themselves, we were not able to identify influences on the draft. Future research could investigate the creation of the draft guideline by WHO’s Nutrition Guidance Expert Advisory Group’s Subgroup on Diet and Health and the nature of the initial public consultation. Second, automated content analysis rests on the assumption that word choice reflects an underlying ideology. However, industry may seek to mimic the language of WHO or, conversely, United Nations’ agencies such as WHO may adopt the language of industry. This effect has been observed in textual analyses of the World Bank’s language, which evolved over time to correspond to that of the banking sector.^29^ This mimicking may, in part, account for the relatively close association observed between the text of WHO’s sugars intake guideline and the language used by industry. Inevitably, WHO’s guideline is cited by other organizations and industry, in particular, may cite it in an effort to refute it – this may also partly account for the close overall correspondence between the industry’s and WHO’s positions. Third, it is more difficult to prove there was no influence rather than an adverse influence. Nonetheless, our findings provide some reassurance that the extensive measures taken to ensure that WHO’s guideline-making process is resistant to industry pressure have been effective. In contrast, vested interests have been successful in influencing policy made by the European Union and national legislatures.^15^^,^^30^^,^^31^

Some aspects of WHO’s policy-making process may protect it against industry influence. First, WHO is exclusively concerned with health. Consequently, there is less scope for industries that produce unhealthy commodities and organizations acting on their behalf, including some governments, to divert attention to competing areas such as trade. For example, in national governments and supranational bodies such as the European Union, health may have to compete with other concerns. Second, WHO commissions independent systematic reviews of the evidence, which are more carefully scrutinized for conflicts of interest than reviews commissioned by peer-reviewed journals. The European Food Safety Authority, for example, bases its guidelines on scientific opinion rather than following the same approach to guideline development as WHO.

Many of the sugar industry’s arguments were characteristic of denialism, which is widely practiced by the tobacco and alcohol industries to thwart effective public health interventions.^32^ The overarching strategy was to promote doubt and, thereby, undermine the case for changing the status quo. Several methods were employed. One was to attempt to confuse: the relationship between sugar and health outcomes was complicated by, for example, discussing different types of sugar and whether added or total sugar was more important. Another was to set unrealistic expectations for scientific research – the results of observational studies were discounted by arguing that confounding was “certain”. For example, the tobacco industry contributed to guidance on what was termed “good epidemiology practice”, which involved rejecting observational research that found a relative risk less than 2 – thereby excluding all research on passive smoking.^33^ A third method was to divert attention to other risk factors, such as a lack of physical activity, which excused the sugar industry from responsibility. Moreover, the industry could claim it was promoting physical activity as part of its corporate social responsibility or its commitment to the European Union’s Platform on Diet and Physical Activity. A fourth method, which is widely used by the alcohol industry, was to shift attention from measures to reduce sugar consumption towards measures to avoid harm: for example, the prevention of dental caries by water fluoridation and the use of fluoride toothpaste.

Our findings have important implications. Crucially, WHO’s guideline development process appears robust. This is important in light of ongoing efforts by industry to amend the process, especially because updates to WHO’s guidelines on trans fats and saturated fats are planned. However, it may be prudent to allow greater time for public consultations and to be more explicit from the outset about the quality of the evidence base. In addition, the relative weakness of the evidence cited in the sugars intake guideline underscores the importance of carrying out specific research to provide the type and quality of evidence required to inform the development of public health guidelines. Finally, it is clear that the textual and correspondence analysis employed in our study can play a role in evaluating the independence of the guideline development process, which can vary across United Nations’ agencies and other institutions. It can also help to assess the influence exerted by vested interests, particularly industry-funded organizations.
